# Prevalence of hepatitis delta virus infection among hepatitis b virus surface antigen positive patients circulating in the largest province of pakistan

**DOI:** 10.1186/1743-422X-7-283

**Published:** 2010-10-26

**Authors:** Gulshan Zaidi, Muhammad Idrees, Fayyaz Ahmed Malik, Irum Amin, Muhammad Shahid, Saima Younas, Rashid Hussain, Zunaira Awan, Aaliyah Tariq, Khalida Parveen

**Affiliations:** 1 Department of Biotechnology, University of The Lahore, 1-KM Raiwind Road, Lahore, Pakistan; 2 Division of Molecular Virology, National Centre of Excellence in Molecular Biology, University of the Punjab 87-West Canal Bank Road Thokar Niaz Baig, Lahore, Pakistan; 3 Department of Pathology, Independent Medical College, Faisalabad, Pakistan

## Abstract

**Background:**

Hepatitis delta virus (HDV) and Hepatitis B virus (HBV) co-infection is well known to induce a spectrum of acute and chronic liver diseases which further advance to cirrhosis, fulminant hepatitis and hepatocellular carcinoma (HCC).

**Aim:**

The aim of the present study was to determine the prevalence of hepatitis D virus super-infection among hepatitis B surface antigen (HBsAg) positive individuals in the highly populated province of Pakistan which is not well known.

**Methods:**

Sera samples were subjected to HBsAg and anti-HDV screening and finally anti-HDV and HBsAg positive coinfected samples were used for HDV active RNA confirmation using nested polymerase chain reaction (PCR).

**Results:**

Out of total 200 HBsAg positive samples by rapid device, 96 (48%) were also found reactive for HBsAg using enzyme linked immunosorbant assay (ELISA). Out of these HBsAg ELISA positive samples, 80 (88.8%) were anti-HDV ELISA positive which were then subjected to PCR. The amplification results further confirmed 24 (30%) samples to be HDV RNA positive. HDV super-infection was more common in male patients than female patients (81% VS 19%).

**Conclusion:**

The current study shows a high prevalence rate of HDV-HBV co-infection in Pakistan that tends to increase over time.

## Background

More than 350 million individuals worldwide are Hepatitis B Virus (HBV) carriers and at least 5% of these are co-infected with hepatitis delta virus (HDV) [1]. HDV is a delta agent that is deformed and incomplete RNA virus whose replication and expression is dependent on the presence of HBsAg. HDV is considered to be a sub-viral satellite because it can propagate only in the presence HBV [2]. In association with HBV, HDV produces significantly more severe illness than HBV alone [3]. HDV is now well known to induce a spectrum of both acute and chronic liver diseases [4]. Individuals having HBV-HDV co-infection may have more severe acute disease and higher risks of fulminant hepatitis, cirrhosis and hepatocellular carcinoma (HCC) than those having HBV infection alone [5,6].

Over 15 million people are infected with HDV worldwide and its prevalence in Italy, Eastern Europe and western region of Asia is higher than in the rest of world and appears to be endemic in the Middle East [7]. From Pakistan few studies are available on the co-infection of HDV and HBV from other provinces. For example Qureshi and colleagues [8] showed the effect of Lamivudine on Sero conversion of HBeAg positive cases co-infected with Delta hepatitis in Islamabad. Seetlani et al 2008 estimated the prevalence of hepatitis D in HBsAg (hepatitis B surface antigen) positive patient visiting liver clinics in Karachi. Mumtaz et al 2005 stated the epidemiology and clinical pattern of epatitis Delta virus infection in Pakistan [9]. Zuberi et al 2008 determined the frequency of hepatitis C and D in patients of chronic hepatitis B and the treatment response of hepatitis B in such patients [10].

No study on the dual infection of HDV and HBV is available from Punjab, Pakistan that is the largest province of this country. Therefore, the aim of the current study was to estimate the co-infection of hepatitis D virus in hepatitis B surface antigen (HBsAg) positive patients in the largest province of Pakistan.

## Methods

### Sample collection

A total of 200 sera samples that were found positive for HBV by rapid Immunoblot screening methods were received from 2009 to 2010 that were included in this study. A filled standard form containing subject's demographics and risk behaviors were also received along with each sample. Out of these, 96 samples were found positive for hepatitis B surface antigen (HBsAg) by Enzyme linked Immunosorbant Assay (ELISA) method and were used for anti-HDV ELISA analysis. Out of these, 80 anti-HDV ELISA positive samples were further analyzed by HDV PCR. Total 56 anti-HDV ELISA positive samples were found negative for HDV RNA by PCR. All the samples belonged to different regions of Punjab, Pakistan. A filled standard form containing subject's demographics and risk behaviours were also received along with each sample.

### HBsAg and Anti-HDV Screening

Initially all the patient's sera were checked for HBsAg using an ELISA assay kit (*DRG Instruments*, Germany). Anti-HDV antibody was detected in using 3^rd ^generation Enzyme ELISA (*DIA.PRO, Diagnostic Bioprobes Srl Italy*) kits using the methodology described in the manufacturer's protocol.

### HDV RNA extraction and complementary DNA (cDNA) synthesis

HDV RNA was extracted from 100 μL serum samples using Gentra (Puregene, Minneapolis, MN 55441 USA) RNA Isolation kit according to the manufacturer's protocol. The extracted RNA (10 μL) was reverse transcribed into cDNA with Moloney Murine Leukemia Virus (MMLV) reverse transcriptase (Life Technologies Inc., USA). Briefly, 1 μl of HDV-specific antisense primer (10 pmol/μl) nucleotides 873-896 {5'-CCGCGAGGAGGTGGAGATGCCATG-3'} was mixed with 10 μL of extracted RNA followed by incubation at 70°C in thermal cycler for 10 minutes. Then cooled on ice for 2 minutes and added 9 μL of RT Mix that contained 50 mM Tris-HCl (pH 8.3), 7.5 mM KCl, 3 mM MgCl_2_, 0.1 M DTT, 10 mM dNTPs and 200 U of MMLV reverse transcriptase enzyme. cDNA was synthesized at 37°C for 50 minutes and then heat inactivated the MMLV Enzyme at 95°C for 3 minutes. Spun down and stored at -20°C till was used in nested PCR.

### Qualitative detection of HDV cDNA

The qualitative detection of HDV-cDNA was carried out by nested PCR using four μl of synthesized HDV-cDNA. The first round PCR was done in a tube containing 20 μl of PCR reaction mixture (2.5 mM MgCl2, a 100 μM concentration of each of the four deoxynucleotides (dNTPs), 10 pM of each outer sense nucleotides 695-718 {5'-CATGGTCCCAGCCTCCTCGCTGGC-3'} and outer antisense primers nucleotides 873-896 {5'-CCGCGAGGAGGTGGAGATGCCATG-3'} [11] and 1 U of Taq DNA polymerase Enzyme). The thermocycler (ABI PCR system 2700; PE Applied Biosystem Inc., USA) was programmed to initially incubate the samples for 2 min at 95°C, followed by 35 cycles consisting of 95°C for 1 min, 64°C for 1 min, and 72°C for 1 min. Second round PCR was performed with the same reaction mix and amplification conditions using inner sense nucleotides 729-748 (5'-CAACATTCCGAGGGGACCGT-3') and inner antisense primers nucleotides 846-865 (5'-GAAGGAAGGCCCTCGAGAACAAGA-3') [11]. Standard precautions to avoid contamination during PCR were taken. Positive controls (HDV ELISA & PCR positive) and negative controls (negative HDV serum and distilled water) were included in each run. Finally the PCR products were electrophoresed on a 2% agarose gel prepared in 1× Tris-borate-EDTA (TBE) buffer, stained with ethidium bromide, and evaluated under UV transilluminator. The sizes of PCR products were estimated according to the migration pattern of a 50-bp DNA ladder (Fermentas Life Sciences). The sizes of the first round PCR products and nested PCR were 202-base pair (bp) and 137-bp respectively.

## Results

**Figure 1:**
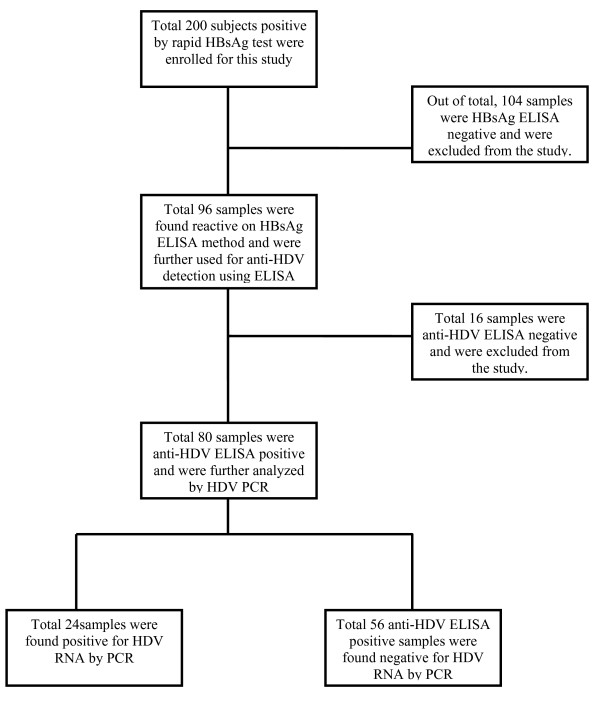
**Patients' Disposition and main features of the study**. During the course of this study, total 200 samples positive by HBsAg rapid test were received from different geographical parts of Punjab province. Out of these, 96 (48%) were found positive by HBsAg ELISA. Of these HBsAg ELISA positive samples, total 80 (83%) samples were found reactive by anti-HDV ELISA method. Of these HBsAg and anti-HDV positive samples, 24 (30%) were further found positive by HDV RNA PCR.

The subject disposition and major features of the results are shown in figure 1. Total 200 samples that were positive in initial screening in different blood banks were received from different regions of the Punjab province. Majority of the samples belonged to males (60.5%) and the mean age of subjects was 42.5 ± 8.9 years. All these subjects were found positive by rapid test device (Accurate or SD Diagnostics) and definitive diagnosis of hepatitis B virus was required by ELISA method. Complete history and records of the patients were maintained. For all the samples HBsAg and anti-HDV ELISAs were carried out at Institute of Molecular Biology & Biotechnology, University of the Lahore, Pakistan. While HDV RNA extraction followed by PCR amplifications for HDV was conducted at the Division of Molecular Virology, National Centre of Excellence in Molecular Biology, University of the Punjab, Lahore. The data sheets showed that the infected patients had been involved in various high risk behaviors such as major minor surgeries, re-use of syringes, dental procedures, blood cupping (in males) etc.

Of the total 200 sera samples, 96 (48%) were found reactive for HBsAg by ELISA method. All the HBsAg positive samples were subjected to anti-HDV ELISA. The result of anti-HDV ELISA showed that 80 (88.81%) out of total 96 subjects were found positive by anti HDV ELISA assay. Of these 80 anti-HDV positive subjects, 35 were females (43.7%) and 45 were males (56.3%).

**Figure 2:**
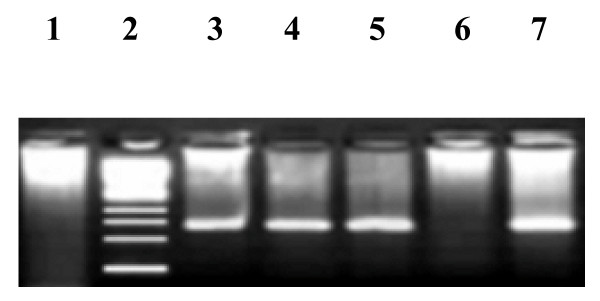
**Gel photograph of different HDV product**. Lane 1: showing no HDV specific band and are thus HDV negative; Lane 2: 50-bp DNA Ladder marker; Lanes 3-5: showing HDV positive samples (137-bp); Lane 6 showing negative control (showing no HDV specific band); Lane 7 is positive control showing 137-bp HDV specific band.

HDV RNA was performed for all 80 anti-HDV ELISA assay for further confirmation of active HDV infection. Figure 2 shows a typical agarose gel showing the specific HDV bands using HDV gene-specific primers in nested PCR. The PCR results confirmed that total 24 (30%) out of 80 anti HDV ELISA positive subjects had active HDV infection in these HBV positive subjects. Of the patients with mixed HBV & HDV infection, 19 were males and 5 were females.

## Discussion

Delta hepatitis is still a major global health problem affecting 15-20 million individuals world wide. HDV co-infection or super infection means that the host liver cells are previously been infected by hepatitis B virus. HDV co-infection or super infection leads to the cirrhosis of the liver and finally hepatocellular carcinoma (HCC) or liver cancer [3,5,6,12]. The current study shows an extremely high prevalence of HDV infection in the province of Punjab Pakistan. Previously it has been assumed that in the rural areas a high prevalence of HDV exists that is not supported by our study as majority of our subjects were from urban areas that means that even in cities a high prevalence rates of dual HDV-HBV infection exists. Though the sample size of our study is not very large as compared to that of earlier studies still the results are very interesting and important as this is the first study from Punjab on the subject where a high prevalence rate of HDV-HBV co-infection has been observed. Importantly we have seen a higher prevalence rate of HDV infection in young males compared to females. Our observation is supported by the findings of a previously published epidemiological survey in Pakistan where the rate of HDV infection was reported higher in young males compared in females [7].

One of the interesting findings of the current study is the observation of high positivity rate of anti-HDV ELISA that is 88.8% in HBV positive patients. This positivity rate is very high as compared to previously reported rates from Pakistan by Mumtaz and colleagues [7], where the reported HDV prevalence rate was 16.6% and Seetlani et al., [9] who showed an overall HDV prevalence of 58.6%. This reported prevalence rate of HDV by Seetlani and co-workers [9] is much higher than that reported by Mumtaz et al. [7], however is still much lower than our findings (about 30%).

The results of this study further suggest that the prevalence of HBV/HDV co-infection in Pakistan has increased during the last decade. On the other hand an overall decline in the worldwide HDV infection has been observed globally [13]. This decrease in HDV prevalence internationally may be due to worldwide HBV vaccination and treatment; for this reason HDV infection is decreased over time along with HBV infection [9,12,14,15]. Even in India that is our neighbor in the East, a decline in HDV infection has been seen in recent years [14]. The same tendency in HDV decline has also been observed in Turkey [15]. Unfortunately in Pakistan we have not seen this tendency in the decline of HDV infection as HBV vaccination programs are not so common and popular in this country as compared to India and Turkey. An important question arise over here that why the prevalence of HDV increase over time? is unknown to us. More research is needed on the subject to find out causes for this increase in the prevalence rate of HDV overtime.

The most interesting finding of the current study is the observation that the rate of active HDV infection is just 30% in Pakistan as was declared by positive qualitative HDV RNA PCR that is still a high rate. Furthermore the prevalence of HDV infection in our PCR study shows that males are more often infected than females. This high rate of HDV infection in males is attributed to risk behaviors. Therefore the general public, health authorities, practitioners and health care managers should be made aware of the risk factors associated with dual infection of HBV and HDV. Proper vaccination and awareness programs should be started to prevent this HBV/HDV dual coinfection.

Our study has few limitations. For example, the sample size is small as only 200 HBsAg positive sera samples by rapid test devices were included in the current study that cannot represent the entire population of Punjab. Next, the information about risk factors was collected from a questionnaire that may vary in completeness and be more exposed to prejudice.

## Conclusion

The results of the current study show a high prevalence rate of HDV-HBV co-infection in Pakistan that has been increased over time. Pakistan is an endemic country for HDV infection. Males are more infected than females. Ministry of health should pay attention to the risk factors responsible for the spread of this dual HDV/HBV infection.

## Abbreviations

HDV: hepatitis delta virus; HBV: hepatitis B virus; HCC: hepatocellular carcinoma; HBSAG: hepatitis B surface antigen; ELISA: enzyme linked immunosorbent assay; CDNA: complementary DNA; RNA: ribonucleic acid; M-MLV RTase: Moloney Murine Leukemia Virus reverse transcriptase; ABI: Applied Biosystems Inc.; PCR: polymerase chain reaction. TBE: tris-boric acid-EDTA; UV: ultraviolet.

## Competing interests

The authors declare that they have no competing interests.

## Authors' contributions

RH, GZ and MI conceived of the study participated in its design and coordination and gave a critical view of manuscript writing. GZ collected samples, epidemiological data, perform all the serological and molecular biology assays and analyzed the data statistically. MI, FAM, ZA, IM, MS SY, AT and RH participated in data analysis. All the authors read and approved the final manuscript.
